# GIGANTEA regulates lateral root formation by modulating auxin signaling in *Arabidopsis thaliana*

**DOI:** 10.1080/15592324.2022.2096780

**Published:** 2022-07-13

**Authors:** Anamika Singh

**Affiliations:** Department of Plant Developmental Biology, Max Planck Institute for Plant Breeding Research, Cologne, Germany

**Keywords:** GIGANTEA, lateral root, red light, auxin, *NAC1*, *TIR1*, *AIR3*

## Abstract

Lateral root (LR) formation is a vital organogenetic process that determines the root architecture in plants. The number of root branches governs the degree of anchorage, efficiency of nutrients acquisition, and water uptake. The molecular pathways involved in LR formation have been extensively studied in *Arabidopsis thaliana* (*At*). A plant hormone, Auxin, is a key regulator of root development and promotes LR formation in plants. A plethora of *Arabidopsis* genes have been identified to regulate LR initiation, patterning, and emergence processes. Recently, the involvement of flowering time control pathways and circadian clock pathways in LR development has come to light, but the connecting link between these processes is still missing. We have established that GIGANTEA (GI), a key component of photoperiodic flowering, can regulate the formation of LRs in *Arabidopsis*. GI is known to be involved in red light signaling and circadian clock signaling pathways. Here, we report that over-expression of *GI* enhances LR formation in red light in *At*. Real-time PCR analysis shows that GI positively regulates the transcription of *TRANSPORT INHIBITOR RESPONSE 1* (*TIR1*) which is an upstream component of auxin signaling. Furthermore, *gi-100* mutant downregulates the LR initiation signaling gene, *AUXIN RESPONSE FACTOR 7* (*ARF7*), and its downstream target gene, *LATERAL ORGAN BOUNDARIES-DOMAIN 16* (*LBD16*). Hence, GI acts as a positive regulator of IAA14-ARF7-LBD16 modules during LR initiation. We have also checked the effect of GI on the expression of *NAC1* and *AIR3* genes which are controlled by TIR1 during LR formation. Our results show that GI induces the *NAC1* transcription and its downstream gene, *AIR3* expression, which leads to the enhancement of LR initiation. Taken together, our results suggest that GI controls the expression of *TIR1* to govern auxin signaling during LR formation in presence of red light and GI can act as a link between circadian clock signaling, flowering time control pathways, light signaling, and lateral root development pathways.

## Highlights

- GIGANTEA enhances lateral root formation in red light.
- Mutations in *GI* cause altered expression of auxin signaling components in presence of red light.
- GI functions upstream of auxin signaling and positively modulates the expression of the Auxin receptor gene, *TIR1*.
- GI positively controls IAA14-ARF7-LBD16 modules during LR initiation.
- Other LR initiation signaling components like *NAC1*, and its downstream target, *AIR3*, are also positively regulated by GI.

## Introduction

Roots are extremely important for anchoring the plant to its growth substrate, enabling water and nutrient uptake from the soil, and sensing environmental signals such as abiotic and biotic stresses.^1,2^ Understanding the molecular mechanisms regulating root architecture in plants is crucial for improving nutrient uptake efficiency and crop yields. Lateral root (LR) formation is an essential organogenetic process that establishes the root architecture system in plants. In *Arabidopsis*, LRs are derived from xylem pole pericycle (XPP) cells positioned within the primary root. These pericycle cells undergo anticlinal, periclinal and tangential cell divisions sequentially to form LR primordium.^2^ The phytohormone, Auxin, is the central regulator of LR development.^3^ Molecular genetic studies have revealed that auxin signaling mediated by transcriptional regulators, Aux/IAAs, and ARFs, are required for LR formation.^3–5^

Global analysis of root transcriptome has identified that several flowering time control genes are expressed in the roots and several flowering genes are differentially expressed during induction of flowering in *Arabidopsis*.^6^ There is a lack of studies to understand the functions of flowering time genes on root development. Only a few data suggest that a shoot-root loop exists to drive sugar/cytokine fluxes and can synchronize root functioning with floral transition.^7^ Similarly, FT-like proteins, regulators of the flowering pathway, are exported from the leaves and induce belowground processes such as bulb formation in onions or tuberization in potatoes.^8,9^

Recent reports show that the circadian clock is rephased during LR formation. Any mutation, suppression or overexpression, in the core circadian clock components exhibits LR emergence defects. Transcripts profiling data showed that nine core circadian clock genes, *ELF3, GI, PRR7, CCA1, LHY, TOC1, PRR5, PRR3*, and *ELF4* oscillate in LR primordia and regulate lateral root formation in plants. Furthermore, the circadian clock genes control the levels of auxin and auxin-related genes such as the auxin response repressor, *IAA14*, and auxin oxidase, *AtDAO2*,^10^ which are the regulators of root development in plants.

Moreover, it was observed that alteration in red light signaling and auxin signaling impair LR formation in *Arabidopsis thaliana* (*At*). The red/far-red ratio (R: FR) which is sensed through phytochromes in the shoot, recruits a signaling transduction cascade that regulates LR formation in the root system.^11^ Although the molecular pathways involved in the regulation of LR formation have been extensively studied, but there is no such report on the connecting link between the circadian clock pathways, flowering time control pathways, and light signaling pathways in the LRs development. In the current study, we explore the plausible mechanism and function of the GIGANTEA, a circadian clock gene, in the regulation of the LR growth in presence of red light.

*GIGANTEA* (*GI*) is an evolutionary conserved plant-specific circadian clock-controlled gene that regulates photoperiodic flowering in plants. GI encodes a nuclear protein that plays pleiotropic functions throughout the developmental stages ranging from seed germination to flowering time control.^12,13^ GI is also involved in red light signaling and controls circadian clock function.^14–16^ Through global root transcriptome analysis, Bouché et al. showed that *GI* was one of the detected genes that were upregulated in roots during the induction of flowering.^6^ Despite the plethora of information regarding the ubiquitous functions of GI, no dedicated study has been undertaken to examine the function of GI in regulating the root architecture system in plants.

## Results and discussion

**Figure 1.**
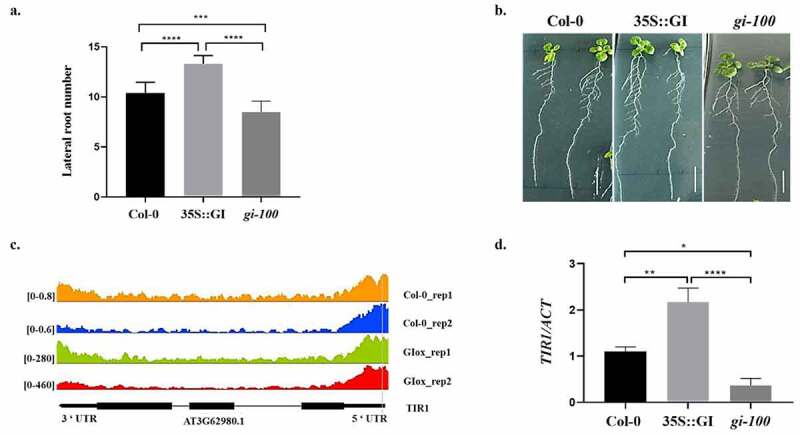
**Effect of GIGANTEA on lateral root development in *Arabidopsis* seedlings**. (a) Graph showing lateral root number in wild type (Col-0), 35S::GI, and *gi-100* seedlings. (b) The phenotype of lateral roots in wild type (Col-0), 35S::GI, and *gi-100* seedlings. All seedlings were vertically grown for 10 d on MS medium under red light, after 8 h of white light induction treatment. Averages of at least 10 seedlings ± S.D. are shown. (c) *TIR1* genome track of read coverage profiles of GI in Col-0 (Orange and blue) and GI-overexpressed (green and red) lines. Peaks represent the sequence enrichment of *TIR1* in Col-0 (Orange and blue) and GI-overexpressed (green and red) lines. Bioinformatic analysis of ChIP-seq data shows GI binds to the promoter region of *TIR1*. Read coverage is presented on the left side of each ChIP-seq. (d) Graph showing the qRT-PCR result of *TIR1* gene expression relative to *ACTIN* in Col-0, 35S::GI, and *gi-100* seedlings which are grown under red light in LD conditions. The qRT-PCR reactions were performed in triplicates. GraphPad Prism software was used to test for significance among the dataset using a one-way analysis of variance (ANOVA) followed by Sidak’s multiple comparisons test (* P < .05, ** P < .01).

To explore the effects of GI on root development, the root phenotype of wild-type Col-0 plants, GI overexpressed under constitutive promoter (35S::GI) lines, and GI T-DNA insertional null mutant, *gi-100*, plants were analyzed. All seedlings were vertically grown for 10 days on MS media under red light at 22°C in LD conditions, after 8 h of white light induction treatment. The number of lateral roots of Col-0, 35S::GI, and *gi-100* seedlings were then counted. Under the red light conditions, the overexpressed GI line, 35S::GI, had more lateral roots density and the *gi-100* mutant showed reduced lateral roots density in comparison to the wild-type Col-0 plants (Figure 1a,b).

After confirming the role of GI in LR development, we wanted to explore the mechanism through which GI regulates root architecture. In a chromatin immunoprecipitation sequencing study by Nohales et al. 2019, putative GI binding target sites were identified by using a pull-down of GI protein from the *Arabidopsis* chromatin preparation.^17^ We did the data mining of the ChIP-seq data available online (GEO: GSE129865) and identified *TRANSPORT INHIBITOR RESPONSE 1* (*TIR1*), an auxin receptor gene, as one of the putative targets of GI. Bioinformatic analysis of ChIP-seq showed an increased read coverage of the *TIR1* gene in case of overexpressed GI as compared to Col-0. TIR1 encodes the TRANSPORT INHIBITOR RESPONSE 1 protein, which contains a series of leucine-rich repeats, a motif called F box and its overexpression promotes auxin response and TIR1/AFB mutants are auxin resistant.^18,19^ The CHIP-seq analysis illustrated that GI has the ability to bind with the promoter region of the *TIR1* gene (Figure 1c). Therefore, to validate the transcriptional regulation of the *TIR1* gene by GI, we have checked the expression of *TIR1* using quantitative RT-PCR in 35S::GI, Col-0, and *gi-100* mutant lines grown under red light conditions. The 35S::GI plants illustrated enhanced *TIR1* expression, while the *gi-100* mutant showed reduced *TIR1* expression in comparison to Col-0 (Figure 1d). Based on these results, we speculate that GI functions upstream of *TIR1* and might bind to the regulatory elements present in the promoter regions of *TIR1* to modulate its expression. This speculation can be further validated by a microarray study by Fornara et al., 2015, which shows that the *gi* mutant in white light has reduced *TIR1* transcripts expression as compared to its WT control.^20^ These results confirm the function of GI in the regulation of *TIR1* expression and suggest that GI can modulate TIR1 downstream signaling pathways which are involved in LR development.

**Figure 2.**
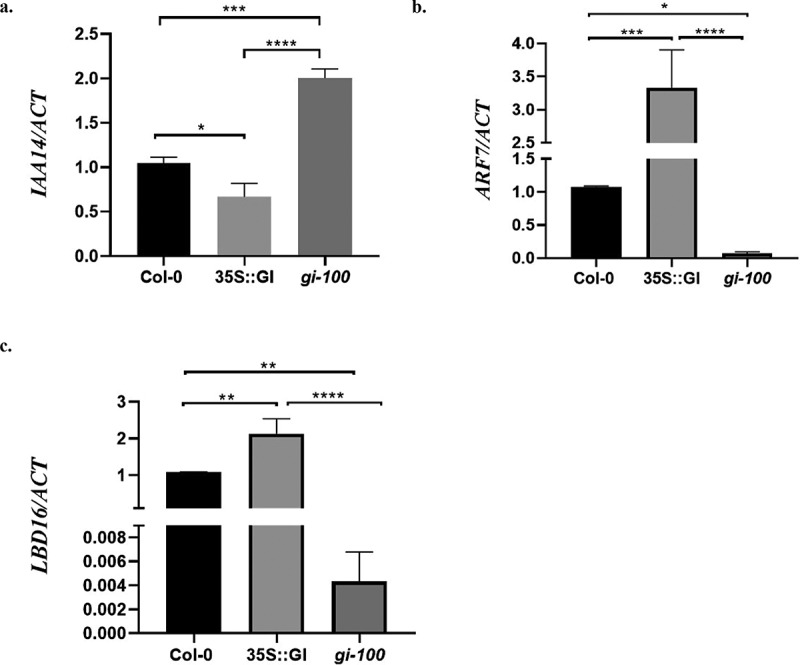
**GIGANTEA modulates auxin signaling by regulating Aux/IAA-ARF modules in *At***. Relative expression of *IAA14* (a), *ARF7* (b), and *LBD16* (c) were analyzed in wild-type (Col-0), 35S::GI, and *gi-100* seedlings by qRT-PCR. All seedlings were grown for 10 d on MS medium under red light, after 8 h of white light induction treatment. Statistical analysis was performed using one-way ANOVA, with significant differences indicated relative to Col-0 (* P < .05, ** P < .01). The qRT-PCR experiments were performed in three biological replicates.

We further wanted to investigate the molecular functions of GI in regulating the developmental events which happen during LR formation like LR initiation. Genetic and physiological studies have shown that the developmental events that happen during LR formation are controlled by phytohormone, Auxin. LR initiation is majorly regulated by LATERAL ORGAN BOUNDARIES-DOMAIN 16 (LBD16) and is under the control of the auxin signaling modules that involve auxin/indole-3-acetic acid (Aux/IAA) transcription repressors and AUXIN RESPONSE FACTOR (ARF) transcription activators.^5,21^ Multiple Aux/IAA-ARF modules such as IAA19/MSG2-ARF7, IAA14/SLR-ARF7 and -ARF19, IAA3/SHY2-ARFs, and IAA28-ARFsTIR1/AFB receptors, act redundantly during LR formation.^4,5,19^ TIR1 acts as an auxin receptor and controls Aux/IAA-ARF modules in the process of LR formation in *Arabidopsis*.^3,19^ Since we confirmed that GI regulates the *TIR1* expression, we further checked the transcripts abundance of *LBD16* and genes involved in Aux/IAA-ARF modules in Col-0, 35S::GI, and *gi-100* plants. Our results demonstrated that the gain-of-function of *GI* suppressed the transcription of auxin/indole-3-acetic acid transcription repressor, *IAA14*, and in turn, its downstream targets *ARF7* and *LBD16* were upregulated in presence of red light. Conversely, *gi-100* mutant showed upregulation of *IAA14*, causing the least expression of *ARF7* and *LBD16* genes as compared to Col-0 and 35S::GI plants (Figure 2a-c). This study elucidated that GI controls the LR initiation process by modulating the expression of *IAA14* and its downstream targets *ARF7* and *LBD16* in presence of red light.

**Figure 3.**
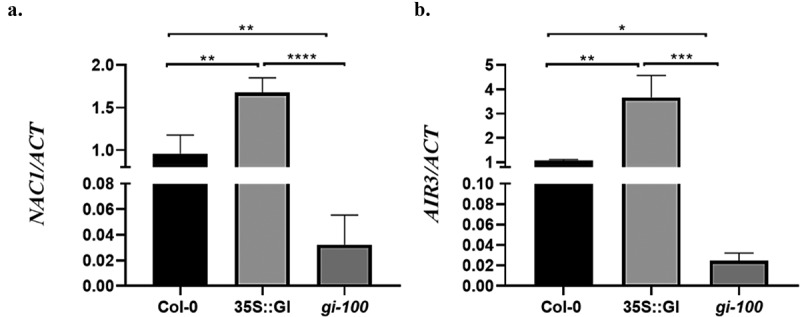
**GI induces *NAC1* and *AIR3* expression in *At***. Transcripts abundance of *NAC1*(a) and *AIR3* (b) were examined by qRT-PCR in wild-type (Col-0), 35S::GI, and *gi-100* seedlings. Samples were harvested from 10 d old seedlings of wild type (Col-0), 35S::GI, and *gi-100* grown under red light in LD conditions. One-way ANOVA was used for statistical analysis, with significant differences indicated relative to Col-0 (* P < .05, ** P < .01). Experiments were repeated three times.

After validating the function of GI in the root initiation process, we further examined its effect on regulating the additional LR signaling components which are regulated by TIR1. Recent studies discovered that NAC1, a member of the NAC family, is a transcription activator that acts downstream of TIR1 and can increase *AIR3* gene expression to upregulate LR formation.^18^ We hypothesized that GI may regulate *NAC1* and *AIR3* gene expression via TIR1 during this developmental process. To test this hypothesis, we analyzed the expression of *NAC1* and *AIR3* in Col-0, 35S::GI, and *gi-100* transgenic lines grown under red light conditions. The GI-overexpressed transgenic line, 35S::GI, shows enhanced transcripts abundance of *NAC1* and *AIR3* as compared to Col-0 in red light. The positive regulation of *NAC1* and *AIR3* expression was further verified by *gi-100* null mutant which showed reduced expression as compared to Col-0 in red light (Figure 3a,b).

In a nutshell, we have shown that GIGANTEA enhances LR formation in red light. Further, we observe that GI functions upstream of *TIR1* and regulates its expression probably by binding to the promoter region. GI modulates the downstream auxin signaling cascade that is regulated by TIR1, including *NAC1* and Aux/IAA-ARF modules, to control LR development in presence of red light.

## Conclusion

**Figure 4.**
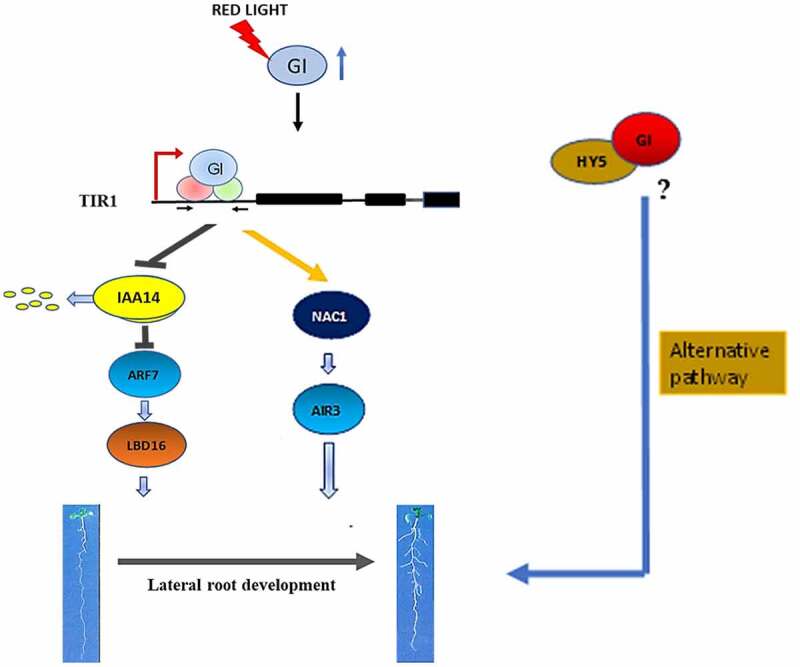
**A Hypothetical model showing regulation of LR formation by GIGANTEA under red light in *Arabidopsis***. Our results suggest that in presence of red light, GI induces the density of lateral roots by modulating Aux/IAA-ARF modules. GI can bind in the promoter region of *TIR1* to induce auxin signal cascade which leads to an increment in *LBD16* gene transcripts during LR initiation. GI can also upregulate *NAC1* expression followed by induction of *AIR3* gene expression which further enhances LRs development in *Arabidopsis*. One probable alternate mode of regulation could be through the transcription factor, HY5. GI could make a complex with HY5 that might regulate LR formation in *Arabidopsis.*

GIGANTEA, a key clock protein, is expressed in the evening and interacts with different kinds of proteins at the transcriptional and post-translational levels to modulate diverse pathways in plants.^12,13,15,16^ GI is a positive regulator of light signal transduction which interacts with Phytochrome B and PIFs during hypocotyl elongation.^14,17^ The gene profiling of the red-light signaling pathway genes in *At* showed that *GI* expression was upregulated in root after red light treatment.^22^ Here, we have investigated the role of GI in LR formation under red light. We have found that GI regulates LR development in *At* via two mechanisms: (1) by regulating Aux/IAA-ARF modules and *LBD16* expression, and (2) by enhancing *NAC1* transcription which further induces *AIR3* transcription, during LR initiation. These two signaling components are regulated by a common modulator, TIR1. Chromatin immunoprecipitation sequencing study by Nohales et al. along with our qRT-PCR analysis suggests that GI might bind to the regulatory sequences of *TIR1* to positively regulate its expression and *TIR1* functions downstream of GI (Figure 4). Although our reports show the *TIR1* mediated function of GI in LR development, there might be alternative mechanisms through which GI can regulate root architecture. One probable alternate mode of regulation could be through the transcription factor, ELONGATED HYPOCOTYL 5 (HY5), which is a light-regulated factor that controls LR formation in *Arabidopsis*.^11^ Reports indicate that HY5 might interact with GI,^17^ so we wonder if GI could make a complex with HY5 that might regulate LR formation in *Arabidopsis*. Therefore, our study provides a novel insight into the role of GIGANTEA that involves TIR1-mediated lateral root formation under red light in *Arabidopsis*.

## Material and methods

### Plant lines and growth conditions

*Arabidopsis thaliana* lines used in this study were Columbia wild type (Col-0), 35S::GI, and *gi-100*. Seeds were surface sterilized by washing them in 70% ethanol and 100% ethanol for 5 min each followed by air drying under a laminar hood. Sterilized seeds were sown on sterile MS media and then transferred at 4°C in the dark for 4 d for stratification. The seeds were then vertically grown for 10 d on MS medium under red light at 22°C in LD conditions, after 8 h of white light induction treatment. For counting the lateral root number of seedlings, the MS plates containing seedlings were scanned and the images were obtained with an Epson L220 scanner. Images were examined using Image J software. The data presented were the mean of 10 individual plants. The pattern of the result presented was similar in all three biological replicates.

### RNA extraction and real-time PCR

The qRT-PCR was performed as published previously with minor modifications.^23^ For RNA isolation, ~50 mg of *At* seedlings after were harvested with liquid nitrogen in the same light condition. Total RNA was isolated using Qiagen Plant RNeasy mini kit followed by cDNA preparation from 1 μg of RNA of each sample using Bio-Rad iScriptTM Reverse Transcription Super-mix. The quantitative RT-PCR (qRT-PCR) was performed using the CFX384 TouchTM Real-time detection system (Bio-Rad Laboratories), following the manual of iTaqTM universal SYBR green supermix. Gene-specific primers were designed using the Primer Quest tool. All reactions were carried out in Hard-shell 384-well PCR plates (provided by Bio-Rad, Cat #: HSP3805) Table 1. The PCR mix and thermocycler program for the qPCR were similar to those done by Singh, 2022.^23^ Transcripts level were normalized with *ACTIN*. Each qRT-PCR reaction was performed in three biological replicates and all data were presented as mean ± SEM.

**Table 1.** List of primers used in qRT-PCR.

GENES	Forward Primer	Reverse Primer
*TIR1*	TAATTTGGTACCTGACGGATGG	CACCATCCTCTTCAGCCTTATC
*IAA14*	CCTTCTAAGCCTCCTGCTAAAG	CTTCGCCGCTCTTCTGATTA
*ARF7*	CCCTCCAAGTTACTGAGCTTTC	GCTGAGGCAACTGAGACATT
*LBD16*	TCAAACCGGAGGAGGAGTAT	AGCCTGAAGCTCACCTAAATC
*NAC1*	GGATCTCATCATCCTCCCAATC	CAGCTTCCCATGTTGTCTCT
*AIR3*	GGATGACATTCCTGGACCTATC	GACCGGGATTCACAGCTAAA
*ACTIN*	CGATGAAGCTCAATCCAAACGA	CAGAGTCGAGCACAATACCG

### Statistical analysis

All the statistical analyses used in this study were performed using GraphPad Prism software version 8.0.1. One-way ANOVA analysis with multiple comparisons (Sidak) was used to analyze column graphs.
